# Studies on Prion Replication in Spleen

**DOI:** 10.1155/2001/95404

**Published:** 2001

**Authors:** Alex J. Raeber, Fabio Montrasio, Ivan Hegyi, Rico Frigg, Michael A. Klein, Adriano Aguzzi, Charles Weissmann

**Affiliations:** 1 lnstitute of Neuropathology University Hospital Zürich 8091 Switzerland; 2 lnstitute of Molecular Biology University of Zürich Zürich 8057 Switzerland; 3 Cytos Biotechnology AG Wagistr. 21 Zürich-Schlieren 8952 Switzerland; 4 MRC Prion Unit/Neurogenetics Imperial College School of Medicine at St Mary's Norfolk Place London W2 1PG UK

**Keywords:** FDC, lymphoreticular system, prion propagation, scrapie, transgenic mice

## Abstract

Some of the early events following scrapie infection take place in the lymphoreticular system (LRS) and result in significant replication of prions in lymphoid organs. The identity of the cells in the LRS that produce prions and their role in neuroinvasion are still unknown. We find that in the spleen of scrapie-infected mice, prions are associated with T and B cells and to a somewhat lesser degree with the stroma, which contains the follicular dendritic cells (FDC's); curiously, no infectivity was found in lymphocytes from blood of the same mice. Thus, splenic lymphocytes either replicate prions or acquire them from another source. Studies on PrP knockout mice with ectopic expression of PrP restricted to only B or T lymphocytes suggest that neither of these by themselves are competent for prion replication. To determine whether B and T cells are able to pick up prions from other sources, irradiated wild-type mice were reconstituted with PrP-deficient lymphohaematopoietic stem cells. Following intraperitoneal inoculation of these mice, no infectivity was found on splenic lymphocytes whereas the stroma (comprising the radiation-resistant, PrP-expressing FDC's) contained prions. These results imply that splenic lymphocytes can acquire prions, possibly from FDC's, but only if they express PrP.


*Developmental Immunology*, 2001, Vol. 8(3-4), pp. 291-304
Reprints available directly from the publisher
Photocopying permitted by license only

© 2001 OPA (Overseas Publishers Association)
N.V. Published by license under
the Harwood Academic Publishers imprint,
part of The Gordon and Breach Publishing Group,
a member of the Taylor and Francis Group

### Studies on Prion Replication in Spleen

ALEX J. RAEBER[a*], FABIO MONTRASIO[a], IVAN HEGYI[a], RICO FRIGG[a], MICHAEL A. KLEIN[a], ADRIANO AGUZZI[a] and CHARLES WEISSMANN[b†]

[a]*Institute of Neuropathology, University Hospital, 8091 Zürich and* [b]*Institute of Molecular Biology, University of Zürich, 8057 Zürich, Switzerland*

Some of the early events following scrapie infection take place in the lymphoreticular system (LRS) and result in significant replication of prions in lymphoid organs. The identity of the cells in the LRS that produce prions and their role in neuroinvasion are still unknown. We find that in the spleen of scrapie-infected mice, prions are associated with T and B cells and to a somewhat lesser degree with the stroma, which contains the follicular dendritic cells (FDC's); curiously, no infectivity was found in lymphocytes from blood of the same mice. Thus, splenic lymphocytes either replicate prions or acquire them from another source. Studies on PrP knockout mice with ectopic expression of PrP restricted to only B or T lymphocytes suggest that neither of these by themselves are competent for prion replication. To determine whether B and T cells are able to pick up prions from other sources, irradiated wild-type mice were reconstituted with PrP-deficient lymphohaematopoietic stem cells. Following intraperitoneal inoculation of these mice, no infectivity was found on splenic lymphocytes whereas the stroma (comprising the radiation-resistant, PrP-expressing FDC's) contained prions. These results imply that splenic lymphocytes can acquire prions, possibly from FDC's, but only if they express PrP.

*Keywords:* FDC, lymphoreticular system, prion propagation, scrapie, transgenic mice

### INTRODUCTION

The major clinical and pathological manifestations of prion diseases are found in the central nervous system. Experimentally, the agent is most efficiently transmitted by intracerebral (i.c.) inoculation into the host. However the natural route of infection is more likely via the gastrointestinal tract, which is believed to be the main gateway for the agent in sheep scrapie, kuru, BSE (Anderson *et al.*, 1996; Wilesmith *et al.*, 1992) and likely new variant form of Creutzfeldt-Jakob disease (vCJD) (Will *et al.*, 1996). Peripheral infection, e.g. intraperitoneal (i.p.) infection into laboratory rodents likely mimics the natural infection process more closely than i.c. inoculation.

* Corresponding author: A.J. Raeber, Cytos Biotechnology AG, Wagistr. 21, 8952 Zürich-Schlieren, Phone: (+411) 733 4032, Fax: (+411) 733 4019, e-mail: raeber@cytos.com
† Present Address: MRC Prion Unit/Neurogenetics, Imperial College School of Medicine at St Mary's, Norfolk Place, London, W2 1PG, UK

### HOST CELLS IN THE LYMPHORETICULAR SYSTEM INVOLVED IN PRION PROPAGATION

Some of the initial events following scrapie infection take place in the lymphoreticular system (LRS) and result in significant replication of prions in lymphoid organs, prior to neuroinvasion and replication of the agent in the brain (Büeler *et al.*, 1993; Eklund *et al.*, 1967; Fraser and Dickinson, 1970). It is noteworthy that the host fails to mount a classical immune response to the scrapie agent (Kasper *et al.*, 1982), presumably because it consists largely or entirely of a host-derived protein. It is not known which cell types in the lymphoreticular system are targets for the scrapie agent and which, if any, mediate its transport to neural sites of replication.

Studies involving splenectomy (Fraser and Dickinson, 1978), whole body irradiation (Fraser and Farquhar, 1987) and spleen fractionation (Clarke and Kimberlin, 1984) suggested that mitotically quiescent cells located in the stromal fraction of the spleen are involved in propagation of the scrapie agent. It was shown by immunohistochemistry that the protease-resistant isoform of PrP colocalizes with follicular dendritic cells (FDCs) in the spleen of prion-infected mice (Kitamoto *et al.*, 1991; Klein *et al.*, 1998; McBride *et al.*, 1992).

Transport of prions from the periphery to the CNS depends on elements of the LRS, as evidenced by the fact that development of CNS disease after i.p. inoculation with scrapie is impaired or abolished in mice with various forms of immunodeficiency, such as SCID, $RAG^{-/-}$ or μMT mice, while i.c. inoculation continues to be fully effective (Brown *et al.*, 1997; Fraser *et al.*, 1996; Kitamoto *et al.*, 1991; Klein *et al.*, 1997; Lasmezas *et al.*, 1996). Although these studies strongly suggest an important role for lymphoid cells in the spread of the disease from peripheral sites to the brain, there is also evidence for a direct neural spread of the disease from the periphery to the CNS albeit with a lower efficiency (Beekes *et al.*, 1996; Beekes *et al.*, 1998; Kimberlin and Walker, 1986; Kimberlin and Walker, 1989a; Kimberlin and Walker, 1989b; Lasmezas *et al.*, 1996). Little is known about the role of lymphocytes in the propagation of the agent. Fractionation of splenocytes from CJD-infected mice revealed low-density lymphocytes as preferential targets for agent replication. In vitro stimulation of B and T cells with mitogens increased susceptibility to CJD infection (Kuroda *et al.*, 1983). Similarly, mitogenic activation of the host enhanced susceptibility to scrapie infection and conversely, immunosuppressive treatment of mice reduced susceptibility to scrapie infection (Dickinson *et al.*, 1978). Within the framework of the “protein only” hypothesis this could be explained by the fact that mitogenic activation of lymphocytes results in increased surface expression of PrP (Cashman *et al.*, 1990) thereby providing higher levels of substrate for the conversion of $PrP^C$ into $PrP^{Sc}$. Taken together, the available data support a participation of cells of various lymphoid origins in agent propagation and disease pathogenesis.

### LOCALIZATION OF PRION INFECTIVITY IN THE LRS

To determine which cells in the LRS are carriers of prions, we undertook to analyze the levels of prion infectivity in various subsets of lymphoid cells isolated from scrapie-infected mice.

In a first experiment, we analyzed spleens of wild-type (129/Sv-C57BL/6) mice 34 days after i.p. inoculation with RML prions. At this time point, prion levels reach a maximum in the spleen and stay approximately constant during the whole incubation period (Clarke and Haig, 1971). Spleens were fractionated into pulp and stroma. B and T cells, respectively, were purified from the pulp fraction by magnetic-activated cell sorting (MACS), followed by complement lysis of B cells in the T cell fraction and vice versa. Finally, viable cells were isolated by density gradient centrifugation. This three-step procedure led consistently to more than 99% pure T and B lymphocyte preparations, respectively, devoid of detectable cross-contamination, in 5–10% yield. In addition, a non-B, non-T cell population was obtained by depleting splenocytes of B and T lymphocytes by

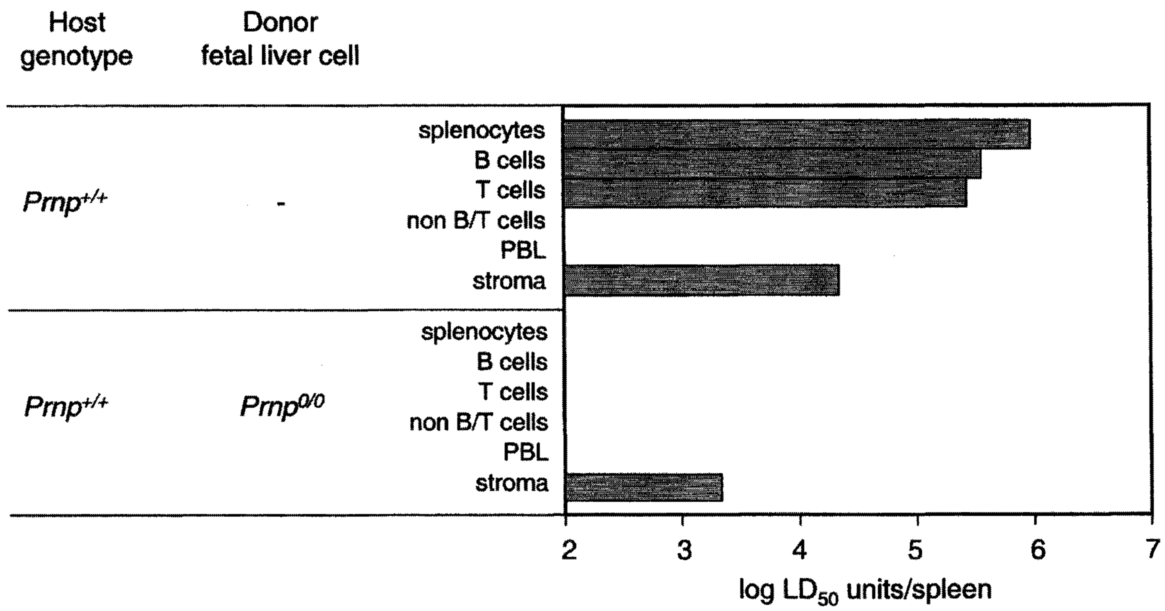

FIGURE 1 Distribution of prion infectivity in spleen fractions and peripheral blood leukocytes of scrapie-infected mice. Serial 10-fold dilutions of splenocytes, splenocyte fractions, spleen stroma homogenate or PBLs were inoculated intracerebrally into groups of four indicator mice and incubation time to terminal scrapie disease was determined. Infectivity titers were calculated from the data given in Raeber et al. (1999a), assuming $3 \times 10^8$ lymphocytes/spleen, of which 65 % were B- and 35 % T-lymphocytes. The detection limit of the infectivity assay corresponds to 100 $LD_{50}$ units per spleen

MACS; this fraction contained < 2% T but no detectable B lymphocytes (Raeber et al., 1999a). The cell preparations and the stroma fraction were analyzed for infectivity by endpoint titration (Brandner et al., 1996; Fischer et al., 1996) (**Figure 1**). Total splenocytes had about 3.5 log $LD_{50}$ units per $10^6$ cells and both B and T cells showed infectivity titers within the same order of magnitude, 3.4 and 3.5 log $LD_{50}$ units per $10^6$ cells, respectively. Strikingly, the non-B, non-T cell population, which consists mainly of monocytes and granulocytes (Binder *et al.*, 1997) contained only about 1 log $LD_{50}$ unit per $10^6$ cells (which could be attributed to the ≤ 2% contamination by T lymphocytes), arguing that prion infectivity in the splenocyte fraction was not due to unspecific contamination with infectivity released from the stromal fraction (Clarke and Kimberlin, 1984). The pulp of a spleen contains 2–4 × $10^8$ cells, about 80% of which are lymphocytes (Binder *et al.*, 1997). Inasmuch as the purified B and T cells were representative of their class as regards infectivity, about 300 × 3.5 log $LD_{50}$ units = 6 log $LD_{50}$ units of infectivity were associated with the pulp and about 4.3 log $LD_{50}$ units with the stroma of one spleen (Raeber *et al.*, 1999a). Essentially all infectivity detected in total spleen extracts was accounted for by the fractions. There was about 50 times more infectivity associated with lymphocytes than with stroma. Clarke and Kimberlin (Clarke and Kimberlin, 1984) have reported about equal distribution of infectivity between pulp and stroma, however their data were obtained with different mouse and scrapie strains than ours.

Because these findings suggested that lymphocytes might be responsible for spreading prions through the organism, we determined the infectivity of peripheral blood leukocytes (PBLs) from the same animals whose spleens had been analyzed. We were surprised that despite the relatively high infectivity associated with splenic B and T cells, intracerebral inoculation of $10^6$ PBLs from buffy coat did not cause disease in any of 4 indicator mice (Raeber *et al.*, 1999a). Because about 80% of murine PBLs are lymphocytes (Binder *et al.*, 1997), less than 1 $LD_{50}$ unit of infectivity was associated with $8 \times 10^5$ lymphocytes; the specific infectivity of peripheral lymphocytes was therefore at least 2–3000 times less that of splenic lymphocytes. Because a mouse has about $2 \times 10^7$ circulating lymphocytes, there would be less than 20 $LD_{50}$ units of cell-associated infectivity, if any, circulating at one time.

Why is infectivity found on splenic but not on circulating lymphocytes? Perhaps only distinct subsets of splenic lymphocytes carry infectivity and these do not enter the blood stream or perhaps prion-carrying splenic lymphocytes are eliminated in the spleen. As regards the role of lymphocytes in the spread of infectivity from the periphery to the CNS, it would seem that circulating, prion-bearing B cells are not required, not only because they were not detected outside the LRS, but because reconstitution of irradiated wild-type mice (Blättler *et al.*, 1997) or immunodeficient mice (Klein *et al.*, 1998) with LRS devoid of PrP restored invasion of the CNS by prions following i.p. inoculation.

These findings demonstrate that splenic lymphocytes are carriers of prions. However, it is not immediately clear whether prions associated with splenic lymphocytes are synthesized *de novo* by these cells or whether splenic lymphocytes are able to scavenge prions from other sources. These might include other cells, which replicate or accumulate prions. Unspecific contamination is unlikely because the specific infectivity of non-B, non-T cells derived from the pulp was 2–3000 times less than that of B or T lymphocytes.

### PRP KNOCKOUT MICE OVEREXPRESSING PRP IN THE LRS

To clarify the role of lymphocytes in prion replication we targeted PrP expression specifically to lymphoid cells in a mouse otherwise devoid of PrP and determined whether such transgenic mice could propagate prions. Using appropriate expression vectors, we targeted PrP expression to three different cell types or tissues in PrP null mice: the lymphoreticular system in general (Raeber *et al.*, 1999b), T lymphocytes (Raeber *et al.*, 1999b) or B lymphocytes (Montrasio *et al.*, 1999) (**Figure 2**).

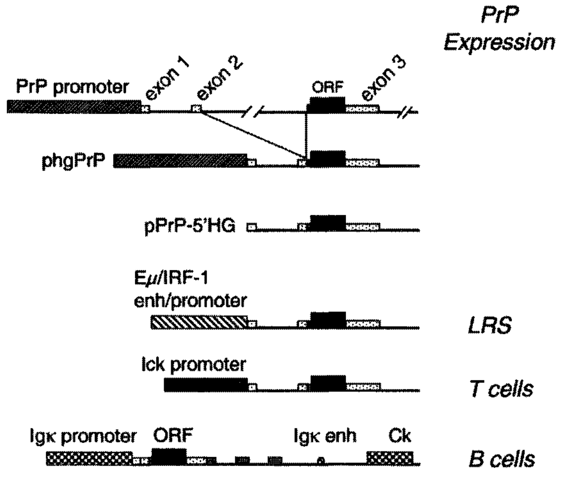

FIGURE 2 Schematic representation of half-genomic PrP transgenes driven by heterologous promoters. The genomic mouse Prnp locus is shown on top. Construction of the "half-genomic" PrP vector (phgPrP) lacking the 12-kb intron 2 has been described. Promoter cassettes were inserted into the promoterless vector pPrP-5'HG to yield plck-PrP-5'HG and pEμ/IRF1-PrP-5'HG. The construct for B cell-specific expression, plgκ-PrP, contained a PrP open reading frame (ORF) driven by the immunoglobulin κ (Igκ) light chain promoter/enhancer

To express PrP ectopically in the LRS we placed the PrP coding sequence under the control of the IRF1-promoter/immunoglobulin heavy chain enhancer (Eμ), which had been shown to lead to overexpression of a linked cDNA in the thymus, spleen and bone marrow (Yamada *et al.*, 1991). Two transgenic *Prnp*$^{0/0}$ mouse lines carrying this construct were established and one of them, Tg94/IRF, was analysed for transgene expression. PrP expression in the spleen of Tg94/IRF mice was more than 1000-fold higher than in wild-type spleen and PrP in brain was 0.05 of that in wild-type (Raeber et al., 1999b). Thus, PrP expression in the spleen of the transgenic mice was more than 20-fold higher than in brain, in marked contrast to wild-type mice, where PrP levels in brain exceed those in spleen about 100-fold. PrP on the surface of peripheral blood leukocytes, as determined by cytofluorometry (FACS), was about 10-fold higher in Tg94/IRF than in wild-type mice (**Figure 3C**). High levels of PrP were also observed on B and T lymphocytes of Tg94/IRF splenocytes (**Figure 3A**) (Raeber *et al.*, 1999b). Cryosections of spleen from non-infected wild-type, *Prnp*$^{0/0}$ and Tg94/IRF mice were doubly stained for germinal center B cells (with peanut agglutinin, green) and PrP (with PrP antiserum 340, red). In wild-type spleens, PrP was mainly present in germinal centers while in Tg94/IRF spleens it was uniformly distributed over white and red pulp (**Figure 4 upper panel**). In **Figure 4 (lower panel)** consecutive spleen sections were labeled with the FDC-specific antibody M1 (green) and PrP antiserum (red; simultaneous staining did not succeed), again revealing a striking overlap of FDC and PrP staining within germinal centers in wild-type spleens. In Tg94/IRF spleens, FDCs were stained in the germinal centers whereas PrP-specific fluorescence was uniform over the whole section, which is compatible with the FACS analysis showing that B and T lymphocytes expressed PrP and with the assumption that FDCs also expressed PrP (Raeber *et al.*, 1999b).

Wild-type and Tg94/IRF mice hemizygous for the transgene cluster were inoculated i.p. As shown in **Table I**, two weeks after inoculation, spleen extracts from Tg94/IRF mice and wild-type animals had the same titer, about 7 log $LD_{50}$ units/ml 10% homogenate and no infectivity was detected in brain. Six months after inoculation the titers of Tg94/IRF spleen extracts were essentially unchanged, somewhat higher than the value of 6.5 for wild-type spleen and no infectivity was detected in Tg94/IRF brains, as compared to 8 log $LD_{50}$ units/ml 10% homogenate for wild-type. This demonstrates that following i.p. challenge with mouse prions, Tg94/IRF mice maintained high titers of prions in the spleen from two weeks up to six months after inoculation without detectable infectivity appearing in the brain. It is remarkable that despite the >1000 fold overexpression of PrP in the spleen of Tg94/IRF mice, the prion titer in this organ was not higher than that in wild-type mice. This apparent paradox can be explained if the high PrP levels in Tg94/IRF spleen reflected PrP in B and T lymphocytes but not in other spleen cells such as FDCs and prion replication were dependent on the latter. Alternatively, it is possible that the level of PrP is not the limiting factor for the formation of prions. Similarly, at the terminal stage of scrapie, the prion titer in brains of *tga20* mice, which overexpress PrP 5–8 fold, is about the same as in wild-type, although the incubation times for the transgenic and wild-type mice are 60 and 160 days, respectively (Fischer *et al.*, 1996).

TABLE I Scrapie infectivity in spleen and thymus of mice inoculated with high doses of RML prions

| *Genotype*[a] | *PrP expression* | *Spleen titer (log $LD_{50}$/ml 10% homogenate)*[b] | | *Thymus titer (log $LD_{50}$/ml 10% homogenate)*[b] | |
|---|---|---|---|---|---|
| | | *2 weeks* | *6 months* | *2 weeks* | *6 months* |
| *Prnp*$^{+/+}$ | | 7 | 6.5 | 3.5 | 4.5 |
| *Prnp*$^{0/0}$ | | <1.5 | <1.5 | <1.5 | <1.5 |
| Tg94/IRF | LRS | 7 | 7 | nd | nd |
| Tg33/lck | T cells | 1.5 | <1.5 | <1.5 | <1.5 |
| Tg306/Igκ | B cells | <1.5 | <1.5[c] | nd | nd |

All data are from Raeber et al. (1999b).
nd not done.

a. Animals were inoculated intraperitoneally with $10^6D_{50}$ of the RML strain of mouse prions.
b. Bioassay limit of detection: 1.5 log $LD_{50}$/ml.
c. 8 week time point.

TABLE II Scrapie infeclivity in spleen and brain of mice following i.p. inoculation with low doses of RML prions

| *Genotype* | *Inoculum RML strain (log $LD_{50}$)* | *Spleen titer (log $LD_{50}$/ml)*[a] | | | *Brain titer (log $LD_{50}$/ml)*[a] |
|---|---|---|---|---|---|
| | | *2 weeks* | *8 weeks* | *12 weeks* | *12 weeks* |
| *Prnp*$^{0/0}$ | 7[b] | <1.5 | <1.5 | <1.5 | <1.5 |
| *Prnp*$^{+/+}$ | 7[b] | 6.2 | 6.9 | 5.9 | 6.8 |
| *Prnp*$^{+/+}$ | 3.5[c] | 2.5 | 6 | 6 | nd |
| *Tg94/IRF* | 3.5[d] | 2 | 5 | 6 | <1.5[e] |

nd not done.

a. Titers are expressed in log $LD_{50}$ units / ml 10% homogenate.
b. Data from Büeler et al. (1993) Cell 73:1339.
c. Data from Clarke and Haig (1971) Res.vet.Sci. 12:195.
d. Data from Raeber et al. (1999) EMBO J. 18:2702.
e. Measured at 6 months post inoculation.

### PRIONS ARE REPLICATED IN THE LRS

Although high prion titers are found in spleen within few days after i.c. or i.p. inoculation, it is not immediately clear whether this reflects de novo synthesis in the LRS or scavenging of infectious agent generated in brain or derived from the inoculum. Inoculation with low prion doses had shown that net increase of infectious agent resulted in the spleen (Clarke and Haig, 1971), however it could not be excluded that the agent was being synthesized in the brain and transported to the LRS. To resolve this question, we inoculated Tg94/IRF mice i.p. with a low dose of RML prions (3.5 log $LD_{50}$ i.c. units) and analyzed spleen homogenates at various times after infection by endpoint titration. As shown in **Table II**, prion titers in the spleen (in log $LD_{50}$ i.c. units/ml 10% homogenate) rose from 2 at two weeks after inoculation to about 5 after 8 weeks and remained at this level up to 12 weeks (Raeber *et al.*, 1999b). Because a spleen weighs about 100 mg, this represents an increase of 4 logs, showing that prions are replicated in the spleen of intraperitoneally inoculated Tg94/IRF mice and are not due to residual inoculum or import from the brain, which even at 6 months contained no detectable infectivity.

### ARE LYMPHOCYTES ABLE TO REPLICATE PRIONS?

We have shown that the LRS efficiently replicates prions after i.p as well as after i.c. infection. To further dissect the host cell types involved in prion replication in the LRS, we targeted PrP expression to lymphocytes and asked whether infectivity is synthesized in the spleen and thymus of such transgenic mice.

### PRP KNOCKOUT MICE OVEREXPRESSING PRP IN T CELLS

Transgenic mouse lines with PrP expression restricted to T lymphocytes were generated with the T-lymphocyte-specific *Lck* promoter (Chaffin *et al.*, 1990). PrP expression in Tg33/lck mice revealed PrP transcript levels in the thymus at least 50-fold higher than in wild-type (data not shown). Significant levels of PrP mRNA were also found in spleen and kidney. Tg33/lck thymus and spleen had PrP levels that were at least 100-fold and 40-fold higher, respectively, than in wild-type. PrP was undetectable in Tg33/lck brain (Raeber et al., 1999b). The high level of PrP expression on T lymphocytes was confirmed by FACS analysis of Tg33/lck thymocytes (**Figure 3B**) and estimated to be 50-fold higher than in wild-type. No PrP expression was detected in Tg33/lck splenic B lymphocytes whereas splenic T lymphocytes were strongly positive for PrP (**Figure 3A**). Immunohistochemical analysis of Tg33/lck spleens (**Figure 4, lower panel**) showed that PrP expression (red) was predominantly in the perifollicular T cell area while the germinal centers, where the FDCs (green) were located showed little red fluorescence over background (Raeber *et al.*, 1999b).

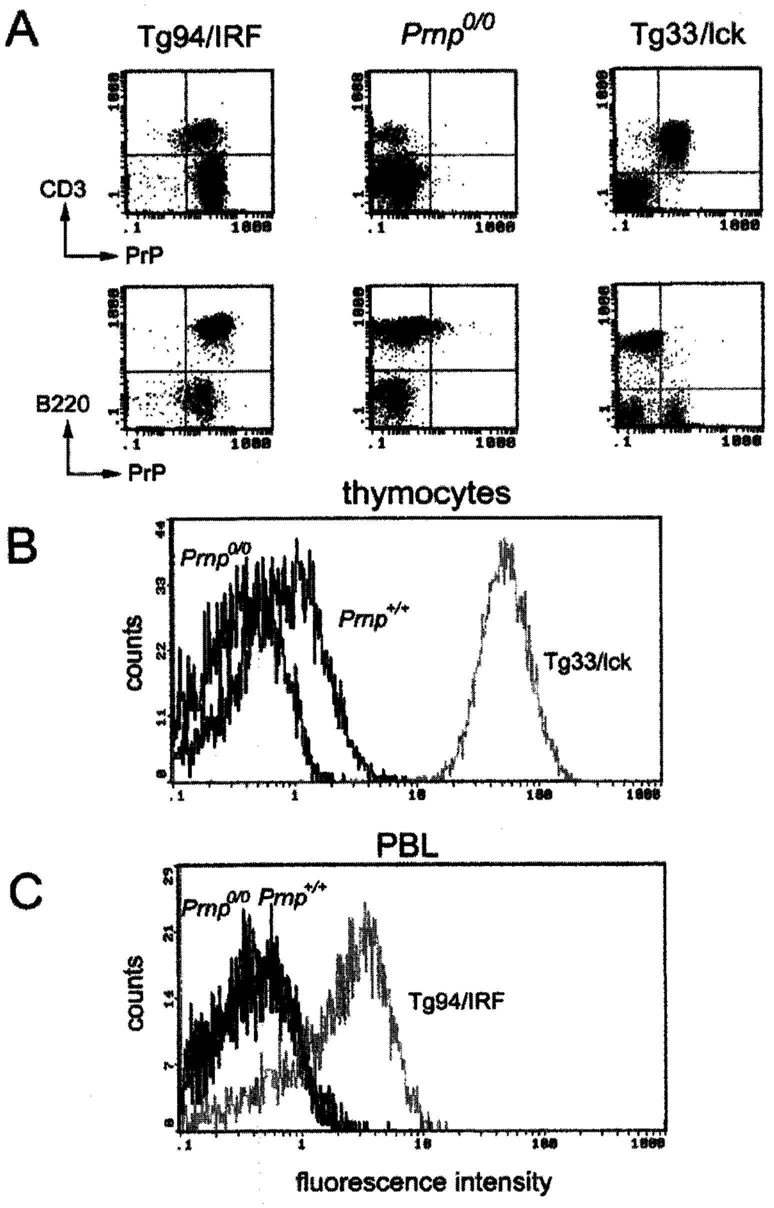

FIGURE 3 Analysis of PrP expression by FACS. Splenocytes (A), thymocytes (B) and peripheral blood leukocytes (PBL) (C) gated for lymphocytes from *Prnp*$^{+/+}$, *Prnp*$^{0/0}$, Tg94/IRF and Tg33/lck mice. Cells were stained with anti-PrP polyclonal antisera R340 and phycoerythrin-conjugated anti-rabbit IgG and analyzed by FACS gated for lymphocytes. For two-colour FACS analysis (A), PrP staining was followed by B cell staining with FITC-conjugated anti-B220 antibodies or T-cell staining with FITC-conjugated anti-CD3 antibodies. From Raeber et al. (1999b)

To determine whether PrP$^{C}$ expression in T lymphocytes of Tg33/lck mice enabled prion replication in thymus and spleen, we assayed tissue extracts pooled from two animals sacrificed at 2 weeks, and 6

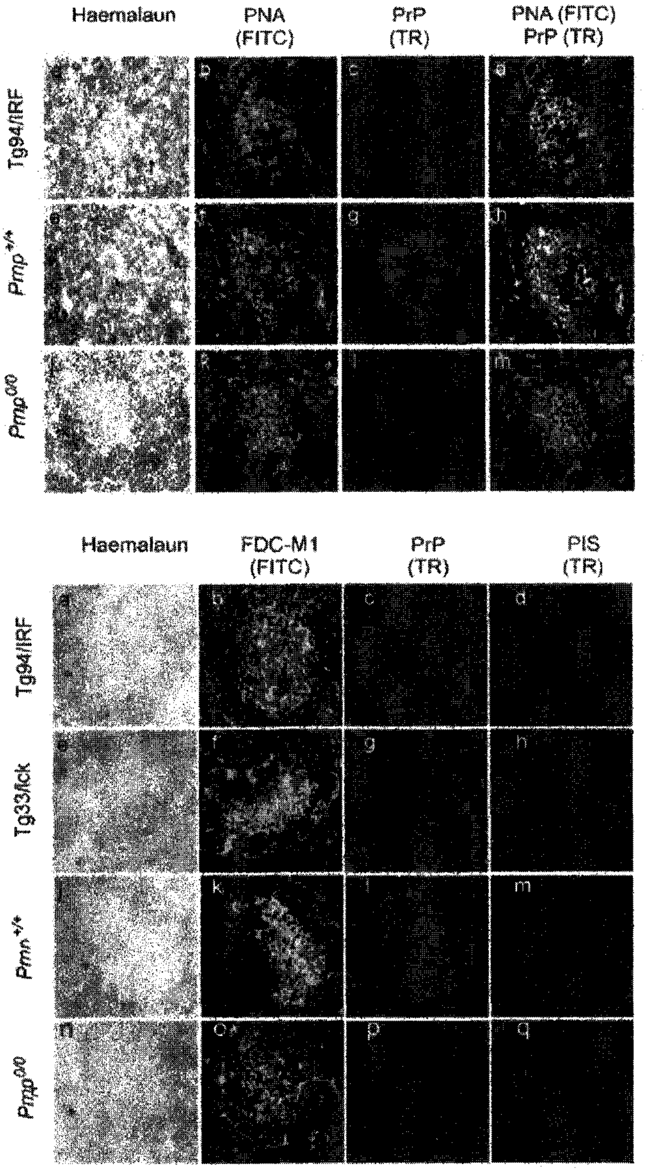

FIGURE 4 Analysis of PrP expression by immunohistochemistry. (UPPER PANEL) Double immunofluorescence analysis of splenic germinal centers in non-inoculated Tg94/IRF (a-d), wild-type mice (e-h), and *Prnp*$^{0/0}$ mice (j-m). Sections were stained with haemalaun (a, e, j), with peanut agglutinin (PNA) (green; b, f, k), and with antiserum R340 to PrP (red; c, g, l). The majority of PNA-labeled germinal center B-cells were PrP-positive in Tg94/IRF mice (d; yellow signal in superimposed images) and in wild-type mice (h), but PrP-negative in *Prnp*$^{0/0}$ mice (m). Original magnification 250x. (LOWER PANEL) Immunofluorescence labeling of follicular dendritic cells and PrP on consecutive sections of spleen from non-inoculated Tg94/IRF (a-d), Tg33/lck (e-h), wild-type mice (j-m) and *Prnp*$^{0/0}$ mice (n-q). Sections were stained with haemalaun (a, e, j, n), antibody FDC-M1 to follicular dendritic cell (green; b, f, k, o), antiserum R340 to PrP (red; c, g, l, p) and rabbit pre-immune serum (PIS) (d, h, m, q). In wild-type spleens (k, l), PrP was stained exclusively in the germinal centers, most strongly in the areas also stained by FDC-M1. In Tg94/IRF mice (b, c), PrP was evenly distributed over the entire section, including the region also stained by FDC-M1. In Tg33/lck spleens, PrP was visualized mainly in the T cell areas but some cells were stained in the region also stained by FDC-M1. No PrP staining above background (q) was found in germinal centers of *Prnp*$^{0/0}$ mice (p). Original magnification 250x. From Raeber et al. (1999b)

months, after i.p. inoculation. In the case of scrapie-infected Tg33/lck mice, homogenates prepared from spleen two weeks after inoculation led to disease in two out of four indicator CD-1 mice corresponding to a titer of about 1.5 log $LD_{50}$ units/ml 10% homogenate. Samples from thymus extracts produced disease in one out of four CD-1 mice giving a titer of less than 1.5 log $LD_{50}$ units/ml 10% homogenate. No infectivity was detected in Tg33/lck spleen or thymus 6 months after inoculation (**Table I**). Thymus homogenates from *Prnp*$^{0/0}$ mice also led to disease in one of four indicator mice. Most likely these borderline infectivities are due to prions persisting from the inoculum (Race and Chesebro, 1998; Sailer *et al.*, 1994). Two weeks and 6 months after i.p. inoculation thymus of wild-type mice had titers of about 3.5 and 4.5 log $LD_{50}$ units/ml 10% homogenate, respectively (Raeber *et al.*, 1999b). Thus even vast overexpression of $PrP^c$ on T lymphocytes, comparable to levels found in wild-type brain, is not sufficient to allow prion replication in thymus or spleen of *Prnp*$^{0/0}$ mice.

### PRP KNOCKOUT MICE OVEREXPRESSING PRP IN B CELLS

It was previously shown that differentiated B cells are crucial for neuroinvasion by prions and for replication of the infectious agent in the LRS (Klein *et al.*, 1997). Whether B cells are directly involved in replicating prions or rather B cell-dependent processes or factors play a role remained uncertain. In order to determine the capability of PrP-expressing B lymphocytes to replicate prions, we generated transgenic mice with PrP-expression restricted to B lymphocytes. The regulatory elements from an Ig kappa variable region gene ($V_k$) were used to drive the transcription of a PrP cDNA cloned with its own initiation codon and leader sequence downstream of the $V_k$ promoter. Nine transgenic founders were established and it was shown by FACS analysis that PrP expression was restricted to B cells with no expression detectable on T cells. Northern blot analysis revealed a complete lack of PrP expression in brain and several other tissues examined. The founder with the highest PrP expression (Tg306/Igκ) showed PrP levels on B cells corresponding to the level found on B cells of heterozygous *Prnp*$^{0/+}$ mice, about 50% of the wild-type. Tg306/Igκ mice were inoculated i.p. with prions and found to be completely resistant to scrapie (Montrasio *et al.*, 1999).

To determine whether $PrP^C$ expression in B lymphocytes of Tg306/Igκ mice enabled prion replication in the spleen, we assayed spleen extracts from animals sacrificed at 2 and 8 weeks after i.p. inoculation. No infectivity was detected in spleens from scrapie-infected Tg306/Igκ mice (**Table I**) whereas prion titers of around $10^6$ $LD_{50}$ units per ml 10% homogenate were found in spleen from scrapie-infected *Prnp*$^{0/+}$ mice sacrificed at 2 and 8 weeks after inoculation (Montrasio *et al.*, 1999). Thus, B lymphocytes alone are not competent for prion replication.

### LYMPHOCYTES LACK A HOST COMPONENT REQUIRED FOR PRION REPLICATION

Using ectopic expression of PrP in B or T cells, we showed that expression of $PrP^C$ on lymphocytes alone is not sufficient to allow prion replication in thymus or spleen of *Prnp*$^{0/0}$ mice. The following explanations can be offered: (1) Lymphocytes are devoid of a conjectural receptor required for prion uptake or lack cellular factor(s) required for prion replication. Several putative PrP receptors have been reported recently (Martins *et al.*, 1997; Rieger *et al.*, 1997; Yehiely *et al.*, 1997), but have not been characterized at the functional level. Experiments with transgenic mice led to the suggestion that a species-specific factor X is required for prion replication (Kaneko *et al.*, 1997; Telling *et al.*, 1995). (2) Lymphocytes are able to replicate prions but are either rapidly eliminated due to a prion-elicited toxic effect or "washed out" as a result of normal turnover. (3) Finally, it is possible that prions administered i.p. in a PrP knockout environment are not transported or transferred to lymphocytes.

### INVOLVEMENT OF STROMAL COMPONENTS IN PRION REPLICATION IN THE LRS

We have shown that prions are replicated in the LRS but that lymphocytes seem to be unable to replicate prions on their own. We have also found that lymphocytes isolated from spleens of scrapie-infected wild-type mice are associated with prions (Raeber *et al.*, 1999a). Thus, it is possible that lymphocytes can either acquire prions from a different cell type or replicate them in dependence of other cells. Because Tg94/IRF mice, which express high levels of PrP on lymphocytes, but also on other cells of the LRS, accumulate prions to high levels in spleen and thymus, PrP expression on other splenocytes appears to be necessary for prion replication in the LRS.

To clarify the role of PrP expression on stromal cells on prion replication in the LRS, we generated mice whose bone marrow was chimeric with regard to PrP expression. We lethally irradiated *Prnp*$^{+/+}$ mice and reconstituted them with fetal liver cells derived from *Prnp*$^{0/0}$ mice. PCR analysis of splenocytes, PBLs, stroma and tail tissue confirmed that these mice had undergone successful reconstitution and FACS analysis of lymphocytes demonstrated the *Prnp*$^{0/0}$ origin of these cells. Spleens from these mice, 34 days after i.p. inoculation with RML prions, were fractionated into pulp and stroma. B and T cells were purified from the pulp fraction by magnetic-activated cell sorting (MACS) followed by complement lysis of B cells in the T cell fraction and vice versa as described above. The cell preparations and the stroma fraction were analyzed for infectivity by endpoint titration. No infectivity was found in either total splenocytes (<1 $LD_{50}$ unit per $10^6$ cells), or in purified B or T lymphocytes (<1 $LD_{50}$ unit per $10^5$ cells). However, prion titers in the stromal fraction were close to those in wild-type mice (**Figure 1**) (Raeber *et al.*, 1999a).

In the previous study aimed at localizing prion infectivity in the LRS of wild-type mice, we found prions associated with splenic lymphocytes. We then asked whether association of infectivity with lymphocytes of scrapie-infected wild-type mice was specific or adventitious. Because lymphocytes interdigitate with FDCs (Heinen *et al.*, 1995), they might have acquired prions or prion-containing, torn-off membrane fragments from the latter during the isolation procedure. However, the finding that wild-type mice reconstituted with PrP-less FLCs show no infectivity on splenic lymphocytes but significant prion titers in the stroma argues that if this "transfer" hypothesis is correct, the postulated adhesion of scrapie agent is dependent on the presence of PrP on lymphocytes; PrP would then function as receptor for the infectious agent. But which cell-type in the LRS replicates prions in the first place?

Prion infectivity in the stromal component of peripheral lymphoid organs is thought to reside in radiation-resistant post-mitotic cells (Fraser and Farquhar, 1987; Fraser *et al.*, 1989). A prime candidate is the follicular dendritic cell, because the normal isoform of the prion protein, $PrP^c$ seems to co-localize with FDCs in uninfected mice (McBride *et al.*, 1992) while $PrP^{Sc}$ co-localizes with FDCs in mice inoculated with CJD or scrapie agent (Kitamoto *et al.*, 1991; Klein *et al.*, 1998). Thus, a likely source of prions in the LRS would be the FDCs (Brown *et al.*, 1999).

### A MODEL FOR PRION REPLICATION IN THE LRS

From studies of transgenic mice with ectopic PrP expression and from the analysis of prion titers in splenocytes of wild-type and bone marrow chimeric mice, a picture of the mechanisms of prion replication in the LRS is beginning to emerge (**Figure 5**). Following prion infection via peripheral routes, prions are transported to and gain access to the LRS. It is unclear which cells in the periphery support transport of prions. Macrophages are possible candidates because of their phagocytic activity and because their mobility allows them to circulate between lymphoid organs, blood and the parenchyma of many organs. In lymphoid organs the earliest site of $PrP^{Sc}$ accumulation and perhaps prion replication is the FDC.

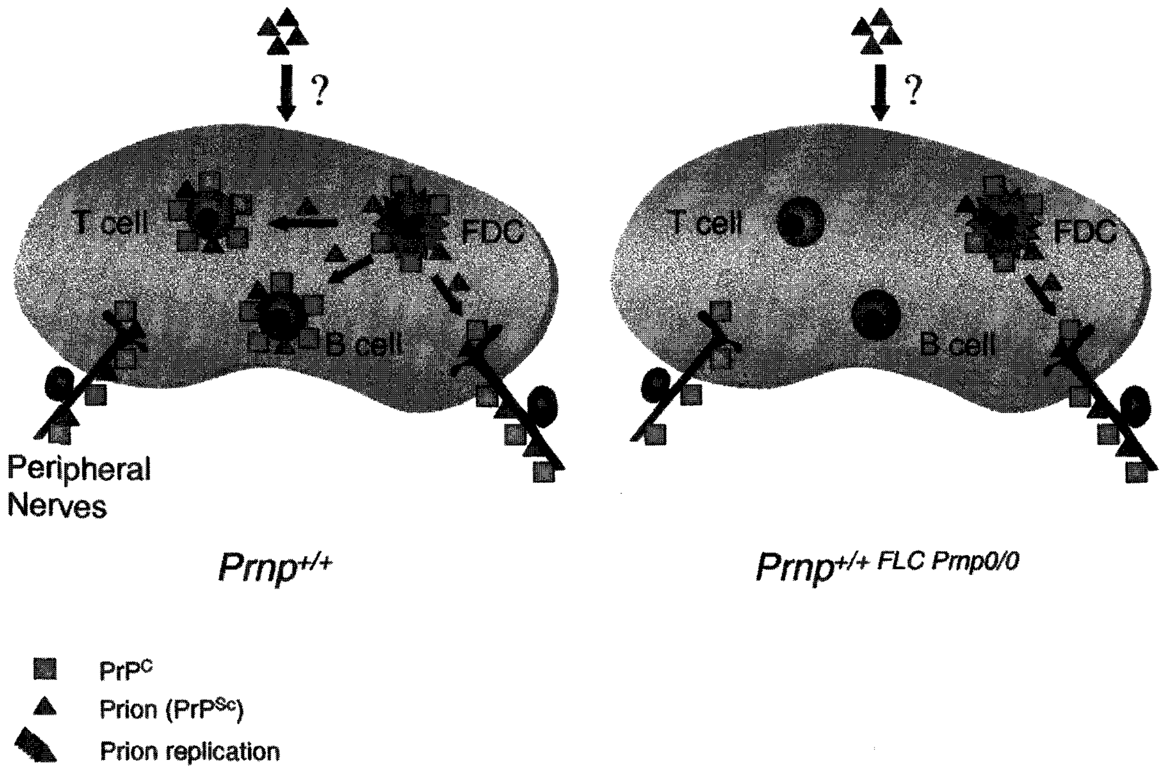

FIGURE 5 Model for prion replication in the LRS. Following prion infection of *Prnp*$^{+/+}$ mice by the intraperitoneal route, prions are found associated with B and T cells as well as with the stromal fraction containing FDCs. Prion replication in these mice likely occurs in FDCs or other stromal cells or within the context of FDC and lymphocytes in the germinal center. Neuroinvasion of prions proceeds via infection of nerve endings of the autonomic nervous system leading to infection of the peripheral and central nervous system where clinical disease develops. In irradiated *Prnp*$^{+/+}$ mice reconstituted with *Prnp*$^{0/0}$ fetal liver cells (FLC), splenic lymphocytes fail to take up prions. But clinical disease in these mice develops with similar kinetics as in *Prnp*$^{+/+}$ mice suggesting that neuroinvasion of prions is not dependent on prion-bearing lymphocytes but rather on the infection of peripheral autonomic nerve terminals

How prions accumulate and might replicate within the context of the FDC network remains elusive. Within the framework of the protein only hypothesis prion replication could occur by conversion of PrP$^{C}$ – expressed on FDCs or neighboring lymphocytes – into further PrP$^{Sc}$ molecules. No prion replication is found in PrP knockout mice expressing PrP only on B or T cells. Therefore, prions found associated with PrP expressing splenic lymphocytes are either acquired from FDCs (in a transfer dependent on PrP) or else synthesized by the lymphocytes but only if they are "infected" by prions presented by FDCs or other stromal cells.

The mechanism by which PrP-expressing lymphocytes acquire prions remains unclear. Since lymphocytes are in close contact with FDCs in the germinal center network it is likely that uptake of prions takes place through direct cellular interaction. Horiuchi *et al.* (1999) have demonstrated binding of PrP$^{C}$ to PrP$^{Sc}$ in vitro, a step that precedes the conversion reaction. To summarize, a wealth of experimental evidence supports an essential role of FDCs in prion replication. Although it was shown that PrP$^{Sc}$ (and possibly prion infectivity) is associated with FDCs, direct evidence for replication of prions in FDCs is still lacking. All of the current model systems and assays to detect prions can not distinguish between accumulation and replication of prions in FDCs. Therefore, it would be important to develop a transgenic mouse model with ectopic PrP expression restricted to FDCs. In addition the PrP transgene could be engineered such that an epitope-tag in PrP would allow to discriminate between *de novo* synthesized $PrP^{Sc}$ and $PrP^{Sc}$ derived from the inoculum.

Following prion replication in the LRS, prions invade the CNS. Theoretically, there are two main possibilities, haematogenous spread or neural spread. The question whether lymphocytes play a role in the spread of prions from the periphery to the CNS is of more than academic interest. The extraordinary lymphotropism of vCJD prions (Hill *et al.*, 1997) raises new public-health concerns and demands urgently to reassess the risk of transmitting vCJD through blood or products thereof derived from individuals suffering from pre-clinical prion disease. Our recent findings suggest that at least in the mouse circulating prion-bearing lymphocytes – and in particular B cells – are not required to physically transport prions to the brain. First of all, prion-bearing lymphocytes were not detected in the blood of scrapie-infected mice although splenic lymphocytes contained significant levels of prion infectivity (Raeber *et al.*, 1999a). More importantly, reconstitution of irradiated wild-type mice (Blättler *et al.*, 1997) or immunodeficient mice (Klein *et al.*, 1998) with lymphohematopoietic stem cells devoid of PrP restored invasion of the CNS by prions following i.p. inoculation. Collectively, these studies support the view that at least in the mouse haematogenous spread of prions does not play a major role in prion neuroinvasion. The essential role of B lymphocytes in sustaining prion replication in spleen and facilitating neuroinvasion may thus not be that of replicating and/or transporting prions, but of activating and maintaining activated FDCs (Fu *et al.*, 1998; Mackay and Browning, 1998; Matsumoto *et al.*, 1997).

Several lines of indirect evidence point to the peripheral nervous system as the crucial compartment that allows prions to get access to the central nervous system (Kimberlin and Walker, 1979; Beekes *et al.*, 1996; Blättler et al., 1997). It is not clear whether prions can replicate in the peripheral nervous system or whether they are simply transported along nerve fibers. Scrapie prions and $PrP^{Sc}$ were found in the peripheral nervous system of a scrapie-sick sheep (Groschup *et al.*, 1996) but so far $PrP^{Sc}$ has not been detected in the autonomic peripheral nervous system. Interestingly, the innervation of lymphoid tissue is at least in part controlled by lymphocytes themselves as both T and B cells secrete nerve growth factor and on the other hand nerve terminals secrete a variety of molecules to stimulate the immune system (Straub *et al.*, 1998).

A thorough understanding of the role of the immune system in peripheral prion pathogenesis is of immediate importance in assessing the risk of iatrogenic transmission of prions and in the development of diagnostic and therapeutic strategies for prion diseases.

### *Acknowledgements*

This work was supported by the Kanton of Zürich, and by grants of the Scweizerischer Nationalfonds to A.J.R., A.A. and C.W. and the European Union to A.A. and C.W.

### *References*

Anderson, R.M., Donnelly, C.A., Ferguson, N.M., Woolhouse, M.E., Watt, C.J., Udy, H.J., MaWhinney, S., Dunstan, S.P., Southwood, T.R., Wilesmith, J.W., Ryan, J.B., Hoinville, L.J., Hillerton, J.E., Austin, A.R. and Wells, G.A. (1996). Transmission dynamics and epidemiology of BSE in British cattle. Nature, **382**, 779–88.

Beekes, M., Baldauf, E. and Diringer, H. (1996). Sequential appearance and accumulation of pathognomonic markers in the central nervous system of hamsters orally infected with scrapie. J. Gen. Virol. **77**, 1925–34.

Beekes, M., McBride, P.A. and Baldauf, E. (1998). Cerebral targeting indicates vagal spread of infection in hamsters fed with scrapie. J. Gen. Virol. **79**, 601–7.

Binder, D., Fehr, J., Hengartner, H. and Zinkernagel, R.M. (1997). Virus-induced transient bone marrow aplasia: major role of interferon-alpha/beta during acute infection with the noncytopathic lymphocytic choriomeningitis virus. J. Exp. Med. **185**, 517–30.

Blättler, T., Brandner, S., Raeber, A.J., Klein, M.A., Voigtländer, T., Weissmann, C. and Aguzzi, A. (1997). PrP-expressing tissue required for transfer of scrapie infectivity from spleen to brain. Nature **389**, 69–73.

Brandner, S., Isenmann, S., Raeber, A., Fischer, M., Sailer, A., Kobayashi, Y., Marino, S., Weissmann, C. and Aguzzi, A. (1996). Normal host prion protein necessary for scrapie-induced neurotoxicity. Nature **379**, 339–43.

Brown, K.L., Stewart, K., Bruce, M.E. and Fraser, H. (1997). Scrapie in immunodeficient mice. Biochem. Soc. Trans. **25**, 173S.

Brown, K.L., Stewart, K., Ritchie, D.L., Mabbott, N.A., Williams, A., Fraser, W.I. and Bruce, M.E. (1999). Scrapie replication in lymphoid tissues depends on prion protein-expressing follicular dendritic cells. Nature Med. **5**, 1308–1312.

Büeler, H.R., Aguzzi, A., Sailer, A., Greiner, R.A., Autenried, P., Aguet, M. and Weissmann, C. (1993). Mice devoid of PrP are resistant to scrapie. Cell **73**, 1339–47.

Cashman, N.R., Loertscher, R., Nalbantoglu, J., Shaw, I., Kascsak, R.J., Bolton, D.C. and Bendheim, P.E. (1990). Cellular isoform of the scrapie agent protein participates in lymphocyte activation. Cell **61**, 185–92.

Chaffin, K.E., Beals, C.R., Wilkie, T.M., Forbush, K.A., Simon, M.I. and Perlmutter, R.M. (1990). Dissection of thymocyte signaling pathways by in vivo expression of pertussis toxin ADP-ribosyltransferase. EMBO J. **9**, 3821–9.

Clarke, M.C. and Haig, D.A. (1971). Multiplication of scrapie agent in mouse spleen. Res. Vet. Sci. **12**, 195–7.

Clarke, M.C. and Kimberlin, R.H. (1984). Pathogenesis of mouse scrapie: distribution of agent in the pulp and stroma of infected spleens. Vet. Microbiol. **9**, 215–25.

Dickinson, A.G., Fraser, H., McConnell, I. and Outram, G.W. (1978). Mitogenic stimulation of the host enhances susceptibility to scrapie. Nature, **272**, 54–5.

Eklund, C.M., Kennedy, R.C. and Hadlow, W.J. (1967). Pathogenesis of scrapie virus infection in the mouse. J. Infect. Dis. **117**, 15–22.

Fischer, M., Rülicke, T., Raeber, A., Sailer, A., Moser, M., Oesch, B., Brandner, S., Aguzzi, A. and Weissmann, C. (1996). Prion protein (PrP) with amino-proximal deletions restoring susceptibility of PrP knockout mice to scrapie. EMBO J. **15**, 1255–64.

Fraser, H., Brown, K.L., Stewart, K., McConnell, I., McBride, P. and Williams, A. (1996). Replication of Scrapie in Spleens of Scid Mice Follows Reconstitution With Wild-Type Mouse Bone Marrow. J. Gen. Virol. **77**, 1935–1940.

Fraser, H. and Dickinson, A.G. (1970). Pathogenesis of scrapie in the mouse: the role of the spleen. Nature **226**, 462–3.

Fraser, H. and Dickinson, A.G. (1978). Studies of the lymphoreticular system in the pathogenesis of scrapie: the role of spleen and thymus. J. Comp. Pathol. **88**, 563–73.

Fraser, H. and Farquhar, C.F. (1987). Ionising radiation has no influence on scrapie incubation period in mice. Vet. Microbiol. **13**, 211–23.

Fraser, H., Farquhar, C.F., McConnell, I. and Davies, D. (1989). The scrapie disease process is unaffected by ionising radiation. Prog. Clin. Biol. Res. **317**, 653–8.

Fu, Y.-X., Huang, G., Wang, Y. and Chaplin, D.D. (1998). B lymphocytes induce the formation of follicular dendritic cell clusters in a lymphotoxin alpha-dependent fashion. J. Exp. Med. **187**, 1009–1018.

Groschup, M.H., Weiland, F., Straub, O.C. and Pfaff, E. (1996). Detection of scrapie agent in the peripheral nervous system of a diseased sheep. Neurobiol. Dis. **3**, 191–195.

Horiuchi, M., Chabry, J. and Caughey, B. (1999). Specific binding of normal prion protein to the scrapie form via a localized domain initiates its conversion to the protease-resistant state. EMBO J. **18**, 3193–3203.

Heinen, E., Bosseloir, A. and Bouzahzah, F. (1995). Follicular dendritic cells: origin and function. Curr. Top. Microbiol. Immunol. **201**, 15–47.

Hill, A.F., Zeidler, M., Ironside, J. and Collinge, J. (1997). Diagnosis of new variant Creutzfeldt-Jakob disease by tonsil biopsy. Lancet **349**, 99.

Kaneko, K., Zulianello, L., Scott, M., Cooper, C.M., Wallace, A.C., James, T.L., Cohen, F.E. and Prusiner, S.B. (1997). Evidence for protein × binding to a discontinuous epitope on the cellular prion protein during scrapie prion propagation. Proc. Natl. Acad. Sci. U S A **94**, 10069–74.

Kasper, K.C., Stites, D.P., Bowman, K.A., Panitch, H. and Prusiner, S.B. (1982). Immunological studies of scrapie infection. J. Neuroimmunol. **3**, 187–201.

Kimberlin, R.H. and Walker, C.A. (1979). Pathogenesis of mouse scrapie: dynamics of agent replication in spleen, spinal cord and brain after infection by different routes. J. Comp. Path. **89**, 551–62.

Kimberlin, R.H. and Walker, C.A. (1986). Pathogenesis of scrapie (strain 263K) in hamsters infected intracerebrally, intraperitoneally or intraocularly. J. Gen. Virol. **67**, 255–63.

Kimberlin, R.H. and Walker, C.A. (1989a). Pathogenesis of scrapie in mice after intragastric infection. Virus Res. **12**, 213–20.

Kimberlin, R.H. and Walker, C.A. (1989b). The role of the spleen in the neuroinvasion of scrapie in mice. Virus Res. **12**, 201–11.

Kitamoto, T., Muramoto, T., Mohri, S., Doh ura, K. and Tateishi, J. (1991). Abnormal isoform of prion protein accumulates in follicular dendritic cells in mice with Creutzfeldt-Jakob disease. J. Virol. **65**, 6292–6295.

Klein, M.A., Frigg, R., Flechsig, E., Raeber, A.J., Kalinke, U., Bluethmann, H., Bootz, F., Suter, M., Zinkernagel, R.M. and Aguzzi, A. (1997). A crucial role for B cells in neuroinvasive scrapie. Nature **390**, 687–90.

Klein, M.A., Frigg, R., Raeber, A.J., Flechsig, E., Hegyi, I., Zinkernagel, R.M., Weissmann, C. and Aguzzi, A. (1998). PrP expression in B lymphocytes is not required for prion neuroinvasion. Nature Med. **4**, 1429–33.

Kuroda, Y., Gibbs, C.J., Jr., Amyx, H.L. and Gajdusek, D.C. (1983). Creutzfeldt-Jakob disease in mice: persistent viremia and preferential replication of virus in low-density lymphocytes. Infect. Immun. **41**, 154–61.

Lasmezas, C.I., Cesbron, J.Y., Deslys, J.P., Demaimay, R., Adjou, K.T., Rioux, R., Lemaire, C., Locht, C. and Dormont, D. (1996). Immune system-dependent and – independent replication of the scrapie agent. J. Virol. **70**, 1292–5.

Mackay, F. and Browning, J.L. (1998) Turning off follicular dendritic cells. Nature **395**, 26–27.

Martins, V.R., Graner, E., Garcia-Abreu, J., de Souza, S.J., Mercadante, A.F., Veiga, S.S., Zanata, S.M., Neto, V.M. and Brentani, R.R. (1997). Complementary hydropathy identifies a cellular prion protein receptor. Nature Med. **3**, 1376–82.

Matsumoto, M., Fu, Y.-X., Molina, H., Huang, G., Kim, J., Thomas, D.A., Nahm, M.H. and Chaplin, D.D. (1997). Distinct roles of lymphotoxin alpha and the type I tumor necrosis factor (TNF) receptor in the establishment of follicular dendritic cells from non-bone marrow-derived cells. J. Exp. Med. **186**, 1997–2004.

McBride, P.A., Eikelenboom, P., Kraal, G., Fraser, H. and Bruce, M.E. (1992). PrP protein is associated with follicular dendritic cells of spleens and lymph nodes in uninfected and scrapie-infected mice. J. Pathol. **168**, 413–8.

Montrasio, F., Cozzio, A., Flechsig, E., Frigg, R., Klein, M.A., Rülicke, T., Raeber, A.J., Aguzzi, A. and Weissmann, C. (1999) Prion protein expression restricted to B lymphocytes does not sustain prion replication in PrP knockout mice. Satellite Symposium on *The Role of Germinal Centers in Prion Diseases*, 1 August, Geneva, Switzerland.

Race, R. and Chesebro, B. (1998). Scrapie infectivity found in resistant species. Nature **392**, 770.

Raeber, A.J., Klein, M.A., Frigg, R., Flechsig, E., Aguzzi, A. and Weissmann, C. (1999a). PrP-dependent association of prions with splenic but not circulating lymphocytes of scrapie-infected mice. EMBO J. **18**, 2702–06.

Raeber, A.J., Sailer, A., Hegyi, I., Klein, M.A., Rulicke, T., Fischer, M., Brandner, S., Aguzzi, A. and Weissmann, C. (1999b). Ectopic expression of prion protein (PrP) in T lymphocytes or hepatocytes of PrP knockout mice is insufficient to sustain prion replication. Proc. Natl. Acad. Sci. U S A **96**, 3987–92.

Rieger, R., Edenhofer, F., Lasmezas, C.I. and Weiss, S. (1997). The human 37-kDa laminin receptor precursor interacts with the prion protein in eukaryotic cells. Nature Med. **3**, 1383–1388.

Sailer, A., Büeler, H., Fischer, M., Aguzzi, A. and Weissmann, C. (1994). No propagation of prions in mice devoid of PrP. Cell **77**, 967–8.

Straub, R.H., Westermann, J., Scholmerich, J. and Falk, W. (1998). Dialogue between the CNS and the immune system in lymphoid organs. Immunol. Today **19**, 409–13.

Telling, G.C., Scott, M., Mastrianni, J., Gabizon, R., Torchia, M., Cohen, F.E., DeArmond, S.J. and Prusiner, S.B. (1995). Prion propagation in mice expressing human and chimeric PrP transgenes implicates the interaction of cellular PrP with another protein. Cell **83**, 79–90.

Wilesmith, J.W., Ryan, J.B., Hueston, W.D. and Hoinville, L.J. (1992). Bovine spongiform encephalopathy: epidemiological features 1985 to 1990. Vet. Rec. **130**, 90–4.

Will, R.G., Ironside, J.W., Zeidler, M., Cousens, S.N., Estibeiro, K., Alperovitch, A., Poser, S., Pocchiari, M., Hofman, A. and Smith, P.G. (1996). A new variant of Creutzfeldt-Jakob disease in the UK. Lancet **347**, 921–925.

Yamada, G., Ogawa, M., Akagi, K., Miyamoto, H., Nakano, N., Itoh, S., Miyazaki, J., Nishikawa, S., Yamamura, K. and Taniguchi, T. (1991). Specific depletion of the B-cell population induced by aberrant expression of human interferon regulatory factor 1 gene in transgenic mice. Proc. Natl. Acad. Sci. U S A **88**, 532–6.

Yehiely, F., Bamborough, P., Da Costa, M., Perry, B.J., Thinakaran, G., Cohen, F.E., Carlson, G.A. and Prusiner, S.B. (1997). Identification of candidate proteins binding to prion protein. Neurobiol. Dis. **3**, 339–55.

